# Coexisting Frailty With Heart Failure

**DOI:** 10.3389/fphys.2019.00791

**Published:** 2019-07-03

**Authors:** Izabella Uchmanowicz, Jadwiga Nessler, Robbert Gobbens, Andrzej Gackowski, Donata Kurpas, Ewa Straburzynska-Migaj, Marta Kałuzna-Oleksy, Ewa A. Jankowska

**Affiliations:** ^1^ Department of Clinical Nursing, Faculty of Health Sciences, Wroclaw Medical University, Wroclaw, Poland; ^2^ Department of Coronary Heart Disease, Institute of Cardiology, Jagiellonian University Medical College, The John Paul II Hospital, Krakow, Poland; ^3^ Faculty of Health, Sports and Social Work, Inholland University of Applied Sciences, Amsterdam, Netherlands; ^4^ Zonnehuisgroep Amstelland, Amstelveen, Netherlands; ^5^ Department of Primary and Interdisciplinary Care, Faculty of Medicine and Health Sciences, University of Antwerp, Antwerp, Belgium; ^6^ Department of Family Medicine, Faculty of Postgraduate Medical Training, Wroclaw Medical University, Wroclaw, Poland; ^7^ Department of Cardiology, Faculty of Medicine, Poznan University of Medical Science, Poznan, Poland; ^8^ Cardiology Department, Centre for Heart Diseases, 4th Military Clinical Hospital in Wrocław, Wrocław, Poland

**Keywords:** heart failure, cardiovascular disease, frailty syndrome, elderly, management, therapeutic concepts

## Abstract

People over 65 years of age constitute over 80% of patients with heart failure (HF) and the incidence of HF is 10 per 1,000 in people aged above 65 years. Approximately 25% of older patients with HF exhibit evidence of frailty. Frail patients with cardiovascular disease (CVD) have a worse prognosis than non-frail patients, and frailty is an independent risk factor for incident HF among older people. Planning the treatment of individuals with HF and concomitant frailty, one should consider not only the limitations imposed by frailty syndrome (FS) but also those associated with the underlying heart disease. It needs to be emphasized that all patients with HF and concomitant FS require individualized treatment.

## Introduction

Frailty is defined as a multidimensional physiological syndrome, which mainly occurs in people aged above 65 years. It is connected with a significant decrease of physiological reserve caused by numerous co-morbidities, influence of stressors, and in general the failure of homeostasis. It is the result of both the processes taking place within the body and the influence of the external environment on human organism. This finally leads to losses of energy, physical and cognitive ability, and health. One of the important factors determining the emergence and manifestation of this syndrome is the reduced physiological reserve of the body (Xue, 2011).

Frailty is a geriatric syndrome meeting at least three of five criteria: weakness (low grip strength), slowness (decreased walking speed), low level of physical activity, exhaustion, and unintentional weight loss (Fried et al., 2001; Clegg et al., 2013). It is caused by multisystem dysregulations, impaired homeostasis, decreased physiologic reserve, and increased risk of morbidity and mortality (Morley, 2015).

Although several definitions of frailty have been proposed, there is still not a single acceptable definition of this condition. One of the first, introduced by Fried and Walston, is that it is “physiological syndrome, characterized by a decrease in the reserves and resistance to stressors resulting from the accumulation of reduced capacity of various physiological systems, which in turn leads to susceptibility to adverse effects” (Fried and Walston, 2003). Another definition of frailty described this syndrome as a “loss of physiological reserve, combined with endocrine disruption and dysfunction of the immune system” (Rockwood et al., 2007).

Two systematic reviews of 21 studies, including 61,500 of the elderly, showed a frailty prevalence of 4.0–59.1% (Collard et al., 2012; Jha et al., 2015). Based on criteria proposed by Fried et al. (2001), it is estimated that 6.9% of the seniors’ population are frail.

It is important to discuss the role of sarcopenia, malnutrition, and cognitive impairment (CI) in frailty. A 3-year prospective observational study by Tamura et al. (2018) showed that the prevalence of frailty and CI increased with aging, whereas the prevalence of sarcopenia is increasing on the plateau after the age of 80. Also, no significant differences were noted in the prevalence of frailty, CI, and sarcopenia between groups differing in terms of coexisting comorbidities (diabetes, hypertension, or hyperlipidemia), with some exceptions, possibly due to high risk individuals with coexisting cardiovascular diseases (CVD).

The study by Gingrich et al. (2019) showed that sarcopenia, frailty, cachexia, and malnutrition were present in 42, 33, 32, and 15% of the patients, respectively (63% had at least one syndrome). These syndromes are characterized by significant weight loss over the last year, which was most evident in malnutrition patients and least noticeable in frail patients, as well as in those with significantly reduced physical performance. It should be emphasized that, according to the newest findings, frail subjects with malnutrition-sarcopenia syndrome (MSS) or malnutrition risk and sarcopenia are at increased risk of long-term all-cause mortality (Hu et al., 2017).

Frailty was shown to occur frequently in patients with HF with the prevalence ranging from 15 to 74%, depending on the studied population and the method of assessment (Altimir et al., 2005; Lupón et al., 2008; McNallan et al., 2013). Its prevalence increases significantly with age, from 3.2% in patients at age 65–70 years to 23.1% among patients 90 years and older (Fried et al., 2001; Newman et al., 2001; Jha et al., 2015). The recent FRAIL-HF study suggests that frailty may affect more than 70% of heart failure (HF) patients over 80 years of age (Vidán et al., 2014; Ponikowski et al., 2016).

It is been observed that there is a high rate of comorbidities, hospitalizations, and deaths in frailty-affected HF patients. Chronic HF is associated with a higher risk of adverse events, including falls, hospitalizations, and deaths, and then with worse prognosis in these patients (Jha et al., 2015).

There are few frailty assessment scales available to objectively evaluate the level of the symptoms and its changes over time. Patients with a high frailty score will benefit from continuous contact with the HF specialist team, more frequent follow-up, and monitoring and individualized self-care support (Ponikowski et al., 2016).

The recommendations of the European Society of Cardiology (ESC), the American Heart Association (AHA), and the Society for Geriatric Cardiology (SGC) emphasize the importance of awareness of the frailty syndrome (FS) in the treatment of patients with HF (Alexander et al., 2007; Ponikowski et al., 2016).

## The Pathogenesis of Frailty

Pathophysiological mechanisms include chronic inflammation and immune activation, with elevated levels of interleukin-6, C-reactive protein, tumor necrosis factor-α, neopterin, and other substances. Increased counts of white blood cells and impaired differentiation of T cells can be observed (Bellumkonda et al., 2017).

Chronic inflammation has detrimental effects on musculoskeletal and endocrine systems, causes anemia, and nutritional dysregulation. Skeletal muscles weakness is mainly caused by sarcopenia, muscular atrophy, changes in α-motor neurons, poor nutrition, growth hormone production, decreased sex hormone and insulin-like growth factor 1 levels, and decreased physical activity (Narici and Maffulli, 2010). Impaired skeletal muscle strength also contributes to osteopenia and osteoporosis leading to pathological fractures. Important element of frailty is also central nervous system involvement (Hogrel et al., 2015). It can be caused by vascular damage, blood-brain barrier dysfunction and leucoaraiosis, leading to cognitive impairment and depression. The term “cognitive frailty” was recently described as impaired executive function, balance disturbances, falls, functional decline, urge incontinence, and disability (Woods et al., 2013). The above changes are accompanied by loss of appetite, malnutrition, nutritional deficiencies, and weight loss (Goisser et al., 2016).

All the above changes are activated by complex multifactorial causes, including genetic and metabolic factors, environmental stressors, and acute and chronic diseases, common in advanced age. They all act as vicious circles accelerating the progress of mental and physical impairment and disability (Fried et al., 2001). On the other hand, progressing frailty causes exhaustion of compensatory mechanisms and decreases resistance to stressors, infections, and neoplasms, leading to increased morbidity and mortality (Chen et al., 2014).

## Definitions of Frailty and Assessment Tools

Frailty is a syndrome leading to adverse outcomes in older people such as disability in activities in daily living (ADL) (Boyd et al., 2005; Gobbens et al., 2012), hospitalization (Fried et al., 2001), nursing home placement (Kojima, 2018), falls (Fried et al., 2001), and mortality (Shamliyan et al., 2013). In addition, frailty is associated with a lower quality of life among older people (Kojima et al., 2016). This figure is influenced by different characteristics of the sample: the mean age of the participants, the ratio between men and women, and the setting where the sample is located. In addition, the prevalence of frailty depends on the approach of frailty (Collard et al., 2012). In general, two approaches of frailty can be distinguished, an unidimensional and a multidimensional approach. The unidimensional approach considers frailty as a physical problem. The definition by Fried et al. (2001) expresses this approach of frailty well: “Frailty is a biological syndrome of decreased reserve and resistance to stressors, resulting from cumulative declines across multiple physiologic systems, causing vulnerability to adverse outcomes” (Fried et al., 2001).

The multidimensional approach of frailty includes not only physical problems but also psychological and social problems that older people can encounter. An example of a definition that fits well the multidimensional approach of frailty is as follows: “Frailty is a dynamic state affecting an individual who experiences losses in one or more domains of human functioning (physical, psychological, social), which is caused by the influence of a range of variables and which increases the risk of adverse outcomes” (Gobbens et al., 2010a).

In line with these two different approaches of frailty, several operational definitions and measurement instruments have been developed. The Cardiovascular Health Study (CHS) Phenotype of Frailty instrument assesses only physical frailty (Fried et al., 2001). It contains five components: unintentional weight loss, weakness (decreased grip strength), poor self-reported endurance, slow gait speed, and low physical activity (Fried et al., 2001). According to Fried et al. (2001), a person is pre-frail as one or two of the components are present and a person is referred to as frail as at least three of five components of the Phenotype of Frailty are present. Many frailty studies have used this instrument for identifying frail older people. Another widely used frailty instrument is the frailty index (FI), which has been developed by Mitnitski et al. (2001) and is based on the Canadian Study of Health and Aging (CSHA) Cumulative Deficit Model.

The FI assesses physical, psychological, and social frailty components and also diseases and items referring to disability in ADL. A relatively new frailty instrument is the Tilburg Frailty Indicator (TFI) (Gobbens et al., 2010c); this instrument is based on an integral conceptual model of frailty (Gobbens et al., 2010b). As well as the FI, the TFI uses a broad approach of frailty. A recent systematic review by Sutton et al. (2016) showed that the TFI has the most robust evidence of reliability and validity among 38 frailty assessment tools.

In a systematic review and meta-analysis, Denfeld et al. (2017) found that frailty affects almost half of people with heart failure. The prevalence was 42.9% among studies using an unidimensional (physical) approach of frailty and slightly higher (47.4%) among studies using a multidimensional approach of frailty. Older people with HF have a higher risk of adverse outcomes such as rehospitalizations (Uchmanowicz et al., 2018) and mortality (Cacciatore et al., 2005). So, the assessment of frailty in this high-risk group is an emergent research priority.

The most commonly used frailty instrument among people with HF was the Phenotype of Frailty (Fried et al., 2001) (*n* = 8), including modified versions, followed by the Comprehensive Geriatric Assessment (CGA) (Rubenstein et al., 1989) (*n* = 4) and the FI by Mitnitski et al. (2001) (*n* = 2) (McDonagh et al., 2018). The aforementioned TFI is so far used in two studies conducted in Poland (Uchmanowicz et al., 2018), demonstrating that social frailty adversely affects the ability to self-care in older people with heart failure. According to McDonagh et al. (2018) assessment of frailty with a validated instrument as part of a holistic treatment plan for older people with HF should be considered.

It is important to identify a frailty measure that will be suitable for clinical and/or research setting. Nowadays, there are a lot of methods for measuring frailty. The determination of which measurement is most appropriate for clinical and/or research applications is currently the subject of intense debate. Theoretically, there is a wide range of tools, such as the Frailty Phenotype, the Deficit Accumulation Index, the Tilburg Frailty Indicator, the Frailty Staging System, the Italian Frailty Index, the Canadian Health and Ageing Clinical Frailty Scale, and the Survey of Health, Ageing and Retirement in Europe Frailty Index. In addition, the Multidimensional Prognostic Index (MPI) in HF patients may be useful (Uchmanowicz et al., 2015; Dent et al., 2016). The fact is that there is an increasing interest in the assessment of frailty, but, to date, there is no frailty instrument validated specifically in the HF population (McDonagh et al., 2018). Also, frailty and fatigue in HF patients can be measured with many scales but the truth is that none of them have been validated and standardized, which seriously preclude a comparative analyses and big data research.

## Frailty and Heart Failure

People over 65 years of age constitute over 80% of patients with HF, and the incidence of HF is 10 per 1,000 in people aged above 65 years (Go et al., 2013). Approximately 25% of older patients with HF exhibit evidence of frailty (Boxer et al., 2008; Dodson and Chaudhry, 2012). Frail patients with CVD have a worse prognosis than non-frail patients, and frailty is an independent risk factor for incident HF among older people (Shinmura, 2016).

Patients with HF are more susceptible to falls and cognitive impairment because of reduced cerebral perfusion, which accelerates the development of frailty and disability (Singh et al., 2014). Frailty is a strong predictor of mortality, rehospitalization, and impaired quality of life in patients with chronic HF (Singh et al., 2014; Uchmanowicz et al., 2014).

Frailty implies a reduced ability to tolerate biological stressors. The condition is associated with circulating inflammatory cytokines and sarcopenia, features that are shared with HF (Bellumkonda et al., 2017). Both frailty and HF are associated with a proinflammatory phenotype, and frailty is most highly associated with cardiovascular dysfunction (Fedarko, 2011; Nadruz et al., 2017). Bellumkonda et al. (2017) suggest that end-organ disease such as HF exacerbates frailty. It was found that in community-dwelling individuals, moderate and severe frailty have an increased risk of incident HF diagnosis (Khan et al., 2013). Reeves et al. (2016) found that frailty is more common with acute decompensated heart failure (ADHF) as compared to those with chronic stable preserved ejection fraction (HFpEF) and reduced ejection fraction (HFrEF). Frailty is promoted by chronic underlying skeletal muscle changes from long-standing HF (Puthucheary et al., 2013; Kitzman et al., 2014).

HF is associated with cellular perturbations, which suggests that HF could be considered as an accelerated form of aging (Dutta et al., 2012; Wohlgemuth et al., 2014). Although patients with symptomatic HF often have cellular and molecular alterations in muscle cell composition, these changes slightly differ from those of normal aging and inflammatory processes. The changes in skeletal muscle in patients with HF are complex and site-specific and are similar to a mixture of chronic deconditioning and inflammation (Goldwater and Pinney, 2015). It is possible that the processes that underlie both frailty and HF could each perturb homeostasis to lead to low-level chronic inflammation (Bellumkonda et al., 2017).

Chronic inflammatory markers are associated with frailty (Darvin et al., 2014). Sterile inflammation occurs also in conditions such as acute ischemia-reperfusion injury or during chronic inflammatory processes evident with HF (Shen et al., 2013).

Aging in humans has been associated with increased, low levels of circulating pro-inflammatory cytokines (Tracy, 2003; De Martinis et al., 2006). Sterile inflammation may result from the breakdown of tissues, such as adipose, skeletal muscle, or cardiomyocytes, whereas chronic, indolent viral infections (e.g., cytomegalovirus) can also lead to chronic inflammation. This may be exacerbated in HF as increased intravascular pressure may result in intestinal congestion, abdominal discomfort, and appetite loss, which may lead to cachexia (Valentova et al., 2016).

The pathobiologies of frailty and HF share several common pathways, particularly a consistent correlation with inflammatory biomarkers (Afilalo et al., 2014; Uchmanowicz et al., 2014; Goldwater and Pinney, 2015). Frailty is associated with increased circulating of TNF-α, IL-6, IFN-γ, and CRP, and these mediators are also elevated in HF patients (Kalogeropoulos et al., 2010; Mann, 2015). This suggests that there could be shared inflammatory pathways that are activated by HF and frailty (Bellumkonda et al., 2017). In addition, immune cells and cytokines, which are known to exert detrimental effects on the arterial wall by promoting atherosclerosis and vascular senescence, also accelerate the aging process and impact body composition, thereby promoting frailty (Afilalo et al., 2014; Uchmanowicz et al., 2014; Goldwater and Pinney, 2015).

Chronic inflammation and associated vascular dysfunction have also recently been linked to HFpEF (Glezeva et al., 2015; Franssen et al., 2016), the most common form of HF in the older adults (Upadhya et al., 2015). Systemic inflammation can also accelerate skeletal muscle apoptosis and promote sarcopenia (Muscaritoli et al., 2010), and this could enhance immobility and cachexia associated with both HF and frailty (Bellumkonda et al., 2017).

Senescent cells that have acquired a senescence-associated secretory phenotype (SASP) can as well cause local and potentially systemic inflammation. SASP might be a key phenomenon in the association between cellular senescence and the development of age-related CVD and (Shinmura, 2016).

DNA damage, impaired autophagy, and mitochondrial dysfunction are biological processes that occur in both aging and HF, and these processes can lead to metabolic dysfunction, cellular senescence, and ultimately cellular necrosis, leading to activation of innate immunity and the production of inflammatory mediators into the circulation. The protein STAT3 limits redox stress and promotes mitochondrial function, and mice lacking STAT3 have increased proinflammatory cytokines and cardiac fibrosis with age (Jacoby et al., 2003; Bellumkonda et al., 2017). Aged mice deficient in the NLRP3 inflammasome exhibit enhanced walk distance and running time as compared to their wild-type controls, suggesting that NLRP3 may enhance inflammation that leads to frailty (Youm et al., 2013), and there is emerging evidence of NLRP3 activation in HF patients (Butts et al., 2015). Bellumkonda et al. (2017) suggest that the NLRP3 inflammasome may be a common pathway by which frailty and HF interact.

A potential unifying model for a common pathophysiological pathway between frailty and HF may be as well impaired mitophagy and mitochondrial dysfunction within cardiomyocytes and skeletal muscle, causing cell death and activation of innate immunity to induce chronic, low-grade systemic inflammation (Bellumkonda et al., 2017).

Summarizing, HF is related to an anabolic-catabolic imbalance in which adaptive neurohormonal mechanisms and autonomic nervous activation fail (Afilalo et al., 2014; Uchmanowicz et al., 2014). In addition to the upregulation of inflammatory cytokines, abnormalities in the GH/IGF-1 axis, cortisol regulation, and insulin resistance are frequently observed in HF-related frailty (Afilalo et al., 2014; Uchmanowicz et al., 2014). However, frailty might predispose to myocardial damage by reducing resistance to stressors such as myocardial ischemia, pressure and volume overload, and arrhythmias, subsequently leading to decompensation and hospitalization (Afilalo et al., 2014; Singh et al., 2014).

## Clinical Consequences of Frailty and Heart Failure

HF has become one of the biggest challenges of modern cardiology. This is primarily a consequence of a constant increase in the incidence of HF in developed countries, which is in turn associated with population aging. According to current estimates, in 2050, more than 40% of Western Europeans will be older than 60 years (Christensen et al., 2009). As the incidence of FS also increases with age, a growing number of HF patients will likely present with concomitant FS.

Frailty is considered one of the most important issues related to human aging and has significant implications for both patients and healthcare systems (Fried et al., 2001). Published evidence shows that frailty is associated with increased risk of falling, loss of functional independence, worse quality of life, more frequent institutionalization, and higher mortality (Boyd et al., 2005; Puts et al., 2005; Ensrud et al., 2007; Al Snih et al., 2009; Chang et al., 2012; Gale et al., 2014).

FS not infrequently imposes physical limitations in the activities of daily living, such as bathing or dressing. Furthermore, concomitant frailty may mask early manifestations of HF. A walking distance or other objective measures of physical performance can be hardly determined in a frail person who does not undertake any physical activity. Additionally, the physical inactivity may contribute to a progressive cardiac damage, which is either not accompanied by severe clinical symptoms or manifests atypically. This may result in a diagnostic and therapeutic delay, and in some cases, an appropriate therapy may be even implemented too late.

Problems with self-care and limited mobility may hinder access of frail persons to healthcare resources, which may result in an inadequate control of therapeutic outcomes and cause a delay in treatment modifications (Renne and Gobbens, 2018).

The quality of life of both HF patients and frail persons is generally low. Multimorbidity, as well as physical, psychological, and social components of frailty, were shown to exert a negative effect on the quality of life (Fortin et al., 2004; de Nóbrega et al., 2009; Bilotta et al., 2010). It is estimated that nearly half of persons from general population suffer from at least one chronic disease. The most common chronic conditions are arthritis, diabetes mellitus, cardiovascular diseases, cancer, and stroke (Renne and Gobbens, 2018). Many people, especially those aged 65 years or older, present with two or more chronic diseases at a time. The multimorbidity is associated with more frequent hospitalizations and emergency department visits, as well as with worse quality of life (van den Bussche et al., 2011; Mujica-Mota et al., 2015). Available evidence suggests that multimorbidity is more common among women than in men (Renne and Gobbens, 2018).

To minimize the impact of aging, people should maintain adequate physical performance and fitness, inter alia through appropriate planning of their physical activity.

## Therapeutic Concepts in Frailty Associated with Heart Failure

Planning the treatment of individuals with HF and concomitant frailty, one should consider not only the limitations imposed by FS but also those associated with the underlying heart disease.

Frailty can be defined as a physical phenotype (Fried et al., 2001) or a multidimensional concept, which does not refer merely to physical functioning but also expands on psychological and social wellbeing. These two distinct concepts of frailty were also reflected in the instruments used to assess the severity of this condition, as well as in a multidisciplinary approach to its management. The phenotype of frailty is a good example of the first approach (Fried et al., 2001). The approach to FS will have a substantial impact on patient management.

Recently, a special emphasis is put on the individualization of HF management. This tailor-made approach seems to be particularly important for some specific patient populations, among them persons presenting with concomitant FS.

Since FS is frequently diagnosed in older persons who present with multiple comorbidities, problems with self-care, and cognitive disorders, its management can be challenging. HF patients with concomitant FS require more attention than those without. The problem seems to be even more complex, since according to literature, the vast majority (over 70%) of patients with HF and concomitant frailty are older than 80 years (Azad and Lemay, 2014).

The authors of the 2016 ESC guidelines for the diagnosis and treatment of HF emphasized that patients with concomitant FS may benefit more if they are managed by specialized multidisciplinary teams, monitored on a regular basis, and provided with an individualized support in self-care (Ponikowski et al., 2016). Aside from being managed by cardiologists, HF patients with concomitant FS should also receive a complex support from nurses, rehabilitation specialists, psychologists and, in selected cases, also social workers. Thus, the therapeutic plan should extend well beyond a medical consultation. Since many older persons have limited access to specialist medical care, a key role in the therapeutic process should be played by primary physicians who know their patients well and may respond promptly to any changes in their health status.

While the diagnosis of frailty in HF has been a subject of many previous studies, published data about the interventions in frailty are quite limited. It needs to be emphasized that all patients with HF and concomitant FS require individualized treatment. However, a few key common areas can be identified that seem to be vital for the management of this group:

1. Improvement of social support, especially support in self-care, which also includes control of treatment adherence. The character of social support covers many aspects of daily functioning with HF, including quality of the relationship, caring for the person, practical issues, and emotional support, ensuring information support. It was shown that the level of perceived social support was largely related to the overall self-care of HF patients. This mainly concerned specific self-care behaviors such as contact with a healthcare professional to report weight gain, reduced fluid intake, take medication, take fluid injections, and exercise regularly. All these activities are essential elements in the self-care of HF patients, in particular, the adherence to medication, which is a key behavior in the treatment of HF and prevention of admission to hospital (Gallagher et al., 2011). Moreover, measures of physical frailty and social support improve prediction of 30-day outcome after an admission for HF (Sokoreli et al., 2019).
2. It was observed that frailty, multimorbidity, obesity, and decreased physical activity as well as mental health status are risk factors for excessive polypharmacy. The Sweden cohort study among 1,742,336 older adults demonstrated that the mean exposition was 4.6 different drugs with range from 4.4 drugs among participants living in the community to 8.2 in nursing homes. The baseline polypharmacy (≥5 drugs) was in 44% and the excessive polypharmacy (≥10 drugs) was in 12%, while during the follow-up, the incidence rate of polypharmacy was 20 and that of excessive polypharmacy was 8 per 100 person-years (Morin et al., 2018). To avoid excessive polypharmacy with its potentially adverse consequences, doctors should carefully check the appropriateness of the drugs used, especially in patients with multifactorial diseases, obesity, and frailty (Rieckert et al., 2018). Reduction of polypharmacy (within a safe margin) through elimination of medications that are not absolutely necessary in the therapeutic process and leaving only those which are vital for the control of clinical symptoms. Such approach should result in better medication compliance and more satisfactory treatment outcomes.
3. Regular resistance and aerobic exercise. The pivotal role of physical activity is currently recognized in many chronic diseases, including chronic HF and cancer. Exercise is also a key component of management in patients with frailty. One study showed that frail persons who participated in a resistance training program for 1 year after hip fracture less often required hospitalization and nursing home admission (Singh et al., 2012). Published evidence suggests that three weekly exercise sessions (each lasting 45–60 min), performed for at least 5 months, are enough to produce a beneficial effect in older frail adults (Theou et al., 2011). Exercise was shown to improve functional performance of frail persons (walking speed, chair stand, stair climbing, and balance) and to reduce depression and the fear of falling (Morley et al., 2013). What is most important, the exercise should always be individually assessed and should take into consideration the type of training (aerobic/resistance/flexibility/balance), its frequency (2–5 days per week), intensity (low/moderate/vigorous), time (20–60 min per day), volume (50–150 min per week), and progression (tolerance and preference), as well as maximal oxygen consumption (VO2) (Zaleski et al., 2016). There is strong evidence indicating the beneficial effects of exercise in elderly which reduce negative symptoms of frailty syndrome, falls episodes, disturbed mental health, impaired cognitive functions, cardiopulmonary dysfunction, and a wide range of musculoskeletal dysfunctions such as balance problems, gait disturbances, poor muscular endurance, and functional capacity (de Labra et al., 2015).
4. An adequate diet including vitamin D supplementation if necessary. Weight loss is a major component of FS (Landi et al., 2010). Calorie supplementation was shown to enhance weight gain and to reduce morbidity and mortality in undernourished older individuals (Milne et al., 2009). Also, HF is often accompanied by eating disorders and the loss of muscle mass, which may eventually lead to cachexia. Nowadays, cachexia is defined as an unintentional weight loss (without concomitant dehydration) of more than 5% within 12 months (or a decrease in BMI <20 kg/m^2^) and presence of at least three of the following criteria: muscle weakness, fatigue, anorexia, low fat-free mass index, and abnormal laboratory parameters of the blood such as elevated concentrations of inflammatory markers (CRP, IL-6), anemia (hemoglobin <12 g/dl), and hypoalbuminemia (<3.2 g/dl) (Okoshi et al., 2017). Cachexia is a systemic process involving most body tissues, i.e., fat-free tissues (including skeletal muscles), adipose tissue (an energy reservoir), and skeleton (whereby it may lead to osteoporosis). Cachexia may be present in up to 5–15% of HF patients, especially those with advanced stages of HF with reduced ejection fraction (von Haehling and Anker, 2014). HF is also frequently accompanied by disorders of calcium-phosphate metabolism resulting from secondary hypoparathyroidism and vitamin D deficiency, both associated primarily with kidney dysfunction. HF patients may also present with elevated levels of TNF-α, which may result in inhibition of calcitriol and vitamin D synthesis. A decrease in vitamin D concentration is associated with enhanced release of renin and hence may perpetuate cachexia. Thus, it is not surprising that vitamin D supplementation during HF treatment gains a growing number of supporters. Research showed that aside from the increase in serum concentration of vitamin D, the supplementation may also contribute to a decrease in the level of aldosterone, a hormone, the level of which is usually too high in HF patients (Witham et al., 2010; Boxer et al., 2013; Sciatti et al., 2016).

Similar recommendations as those presented above were also included in the frailty consensus published by delegates from six major international, European and US societies (Morley et al., 2013). Although this document was not addressed specifically to HF patients, some data can be safely extrapolated on this group. Nevertheless, we need more empirical data about the outcomes of various interventions in patients with HF and concomitant frailty to develop the management strategies that would be most beneficial for this specific population. Irrespective of the intervention type, the management of frailty in HF should extend onto all three domains of human functioning: physical, psychological, and social wellbeing.

## Summary

HF has become one of the biggest challenges of modern cardiology. This is primarily a consequence of a constant increase in the incidence of HF in developed countries, which is in turn associated with population aging. As the incidence of FS also increases with age, a growing number of HF patients will likely present with concomitant FS. Special attention should be paid to the specific components of frailty, such as the sense of being physically unhealthy, lack of social relations and social support, feeling down, and inability to cope with problems, since all these elements, as well as the HF itself, have the biggest impact on the quality of life (Gobbens et al., 2017; Renne and Gobbens, 2018). Therefore, the recommendations of the ESC, the AHA, and the SGC emphasize the importance of awareness of the FS in the treatment of patients with heart failure (Alexander et al., 2007).

## Author Contributions

IU, MK-O, and EJ were responsible for the conception and design, acquisition of data, analysis, and interpretation of data and drafting the initial manuscript and revising it critically for important intellectual content. IU, MK-O, JN, AG, DK, RG, ES-M, and EJ wrote this manuscript. RG, JN, EJ, and IU improved the grammar, syntax, and flow of our manuscripts prior to submission. All authors read and approved the final manuscript.

### Conflict of Interest Statement

The authors declare that the research was conducted in the absence of any commercial or financial relationships that could be construed as a potential conflict of interest.

## Abbreviations

ADHF: Acute decompensated heart failure
ADL: Activities in daily living
AHA: American Heart Association
CHS: Cardiovascular Health Study
CI: Cognitive impairment
CSHA: Canadian Study of Health and Aging
CVD: Cardiovascular disease
ESC: European Society of Cardiology
FS: Frailty syndrome
HF: Heart failure
HFpEF: Heart failure with preserved ejection fraction
HFrEF: Heart failure with reduced ejection fraction
MSS: Malnutrition-sarcopenia syndrome
SASP: Senescence-associated secretory phenotype
SGC: Society for Geriatric Cardiology
TFI: Tilburg Frailty Indicator.
